# Complete Genome Sequence of the Virulent Klebsiella pneumoniae Phage Geezett Infecting Multidrug-Resistant Clinical Strains

**DOI:** 10.1128/MRA.00685-21

**Published:** 2021-12-02

**Authors:** Belinda Loh, Liwei Zhang, Xiaoting Hua, Yunsong Yu, Long Ma, Xiaoqing Wang, Prasanth Manohar, Ramesh Nachimuthu, Willames M.B.S. Martins, Mark A. Toleman, Sebastian Leptihn

**Affiliations:** a Department of Biological Sciences, Xi’an Jiaotong-Liverpool University, Suzhou, China; b Zhejiang University-University of Edinburgh (ZJU-UoE) Institute, Zhejiang University, International Campus, Haining, Zhejiang, China; c Department of Infectious Diseases, Sir Run Run Shaw Hospital, Zhejiang University School of Medicine, Hangzhou, Zhejiang, China; d Medical School, Lishui University, Lishui, China; e Antibiotic Resistance and Phage Therapy Laboratory, School of Biosciences and Technology, Vellore Institute of Technology (VIT), Vellore, Tamil Nadu, India; f Department of Medical Microbiology, Division of Infection and Immunity, Cardiff University, Cardiff, United Kingdom; g College of Medicine & Veterinary Medicine, University of Edinburgh Medical School, Edinburgh, United Kingdom; Loyola University Chicago

## Abstract

Geezett was isolated from hospital sewage in Hangzhou, China, and exhibits lytic activity against clinical isolates of the nosocomial pathogen Klebsiella pneumoniae. The bacteriophage is a myovirus and has a double-stranded DNA (dsDNA) genome 50,707 bp long, containing 79 open reading frames (ORFs).

## ANNOUNCEMENT

**FIG 1:**
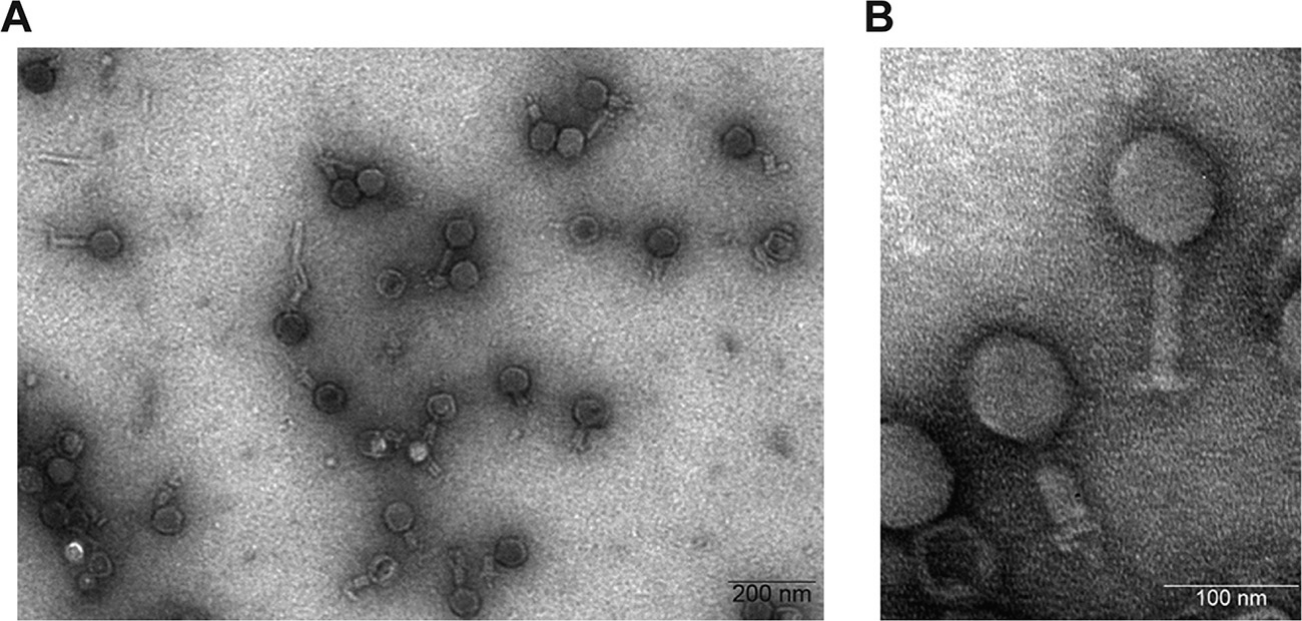
Transmission electron micrograph of Klebsiella pneumoniae phage Geezett. Phages were negative stained using 2% uranyl acetate. (A) Scale bar = 200 nm; (B) scale bar = 100 nm.

In many countries, Klebsiella pneumoniae is a leading cause of hospital-acquired infections (1), which include skin and soft tissue infection, infections of the urinary tract, and also life-threatening bloodstream infections and pneumonia. According to the World Health Organization, the emergence of K. pneumoniae strains resistant to carbapenems and third-generation cephalosporins represents an urgent need for development of new antimicrobial agents such as therapeutic phages (2, 3). Phage Geezett was isolated from sewage water obtained from the Sir Run Run Shaw Hospital in Hangzhou, China, using an enrichment culture of the clinical multidrug-resistant K. pneumoniae strain GZ-1. Characterized primarily by its head-tail structure and a long contractile tail, the phage morphology indicates that it belongs to the *Myoviridae* family of the order *Caudovirales* (Fig. 1).

Phages were obtained from single plaques and amplified prior to DNA extraction, as described previously (4). Phages in the filtrate were used for extracting DNA using the Biomed virus rapid DNA/RNA kit (Beijing, China). Sequencing libraries were prepared using the NEBNext Ultra II DNA library prep kit for Illumina. The phage genome was then sequenced using the Illumina HiSeq platform. A total of 4,404,022 raw reads were obtained with read lengths of 150 bp (paired-end format). The genome coverage was 7,867×. The short-read sequence data were assembled using Unicycler v.0.4.8 (5). Genome annotation and analysis were conducted using default settings via the CPT Galaxy (6) and Web Apollo (7) interfaces. Open reading frames (ORFs) were identified using GeneMarkS v.4.28 (8), GLIMMER v.3 (9), and MetaGeneAnnotator v.1.0 (10) and were manually validated using NCBI BLAST v.2.9.0 searches (11) against the NCBI nonredundant database, the Swiss-Prot database (12), and the Bacterial Virulence Factor Database (VFDB) (13). Default parameters were used unless stated otherwise.

Geezett has a double-stranded DNA (dsDNA) genome of 50,707 bp with a GC content of 48%. It is predicted to encode 79 proteins, of which 23 align to phage genes of known functions. These include proteins involved in transcription regulation, replication, DNA packaging, host lysis, and structural proteins. No genes were found to encode toxins or antibiotic resistance factors. A search for related phages using nBLAST showed that Geezett is novel, with its closest relative being Klebsiella phage vB_KpnM_FZ14 (GenBank accession number MK521906.1), with a sequence coverage of only 66% (at 91.64% nucleotide sequence identity) (14). Several genes are dissimilar, such as the tail spike protein, which has only 68% amino acid sequence coverage with the corresponding protein in phage vB_KpnM_FZ14. The tail fiber protein of Geezett has no similarity with phage vB_KpnM_FZ14; it does, however, have 98% amino acid sequence coverage with that of Klebsiella phage vB_KpnP_KpV48 but with only a 45% amino acid sequence identity, indicating that Geezett might have a different host range compared to other Klebsiella phages.

Lysogeny-related genes and virulence factors were not found in Geezett during genome annotation. The phage is categorized as lytic using the program PhageAI (15), which might allow the deployment of Geezett as a therapeutic phage.

### Data availability.

The complete genome of Geezett has been deposited at GenBank under the accession number MZ504995.1 and the SRA accession number SRR15367659.
